# Impact of Host Genetics and Biological Response Modifiers on Respiratory Tract Infections

**DOI:** 10.3389/fimmu.2019.01013

**Published:** 2019-05-07

**Authors:** Alicia Lacoma, Lourdes Mateo, Ignacio Blanco, Maria J. Méndez, Carlos Rodrigo, Irene Latorre, Raquel Villar-Hernandez, Jose Domínguez, Cristina Prat

**Affiliations:** ^1^Servei de Microbiologia, Hospital Universitari Germans Trias i Pujol, Institut d'Investigació Germans Trias i Pujol, Universitat Autònoma de Barcelona, CIBER Enfermedades Respiratorias, Barcelona, Spain; ^2^Servei de Reumatologia, Hospital Universitari Germans Trias i Pujol, Institut d'Investigació Germans Trias i Pujol, Universitat Autònoma de Barcelona, Barcelona, Spain; ^3^Clinical Genetics and Genetic Counseling Program, Hospital Universitari Germans Trias i Pujol, Institut d'Investigació Germans Trias i Pujol, Barcelona, Spain; ^4^Servei de Pediatria, Hospital Universitari Germans Trias i Pujol, Institut d'Investigació GermansTrias i Pujol, Universitat Autònoma de Barcelona, Barcelona, Spain; ^5^Servei de Pediatria, Hospital Universitari Vall d'Hebron, Vall d'Hebron Institut de Recerca, Facultat de Medicina, Unitat Docent Germans Trias i Pujol, Universitat Autònoma de Barcelona, Barcelona, Spain

**Keywords:** immunogenetics, biological response modifiers, respiratory tract infections, primary immunodeficiencies, inborn errors

## Abstract

Host susceptibility to respiratory tract infections (RTI) is dependent on both genetic and acquired risk factors. Repeated bacterial and viral RTI, such as pneumonia from encapsulated microorganisms, respiratory tract infections related to respiratory syncytial virus or influenza, and even the development of bronchiectasis and asthma, are often reported as the first symptom of primary immunodeficiencies. In the same way, neutropenia is a well-known risk factor for invasive aspergillosis, as well as lymphopenia for *Pneumocystis*, and mycobacterial infections. However, in the last decades a better knowledge of immune signaling networks and the introduction of next generation sequencing have increased the number and diversity of known inborn errors of immunity. On the other hand, the use of monoclonal antibodies targeting cytokines, such as tumor necrosis factor alpha has revealed new risk groups for infections, such as tuberculosis. The use of biological response modifiers has spread to almost all medical specialties, including inflammatory diseases and neoplasia, and are being used to target different signaling networks that may mirror some of the known immune deficiencies. From a clinical perspective, the individual contribution of genetics, and/or targeted treatments, to immune dysregulation is difficult to assess. The aim of this article is to review the known and newly described mechanisms of impaired immune signaling that predispose to RTI, including new insights into host genetics and the impact of biological response modifiers, and to summarize clinical recommendations regarding vaccines and prophylactic treatments in order to prevent infections.

## Epidemiology and Pathogenesis of Respiratory Tract Infections

Acute and chronic respiratory tract infections (RTI) are one of the most frequent causes of infections and antimicrobial prescription, and the leading cause of death in developing countries (1, 2). Pneumonia accounts for 1.3 million deaths annually in children <5 years of age (3). In 2017, 1.6 million people died of tuberculosis (TB). Children (aged <15 years) accounted for 15% of total deaths, higher than their share of estimated cases, suggesting poorer access to diagnosis and treatment. About 1.7 billion people, 23% of the world's population, are estimated to have a latent TB infection (4). The control of latent TB, a stage in which a person is infected with *Mycobacterium tuberculosis* plays an important role in disease control, since dormant bacilli are a reservoir of potential TB cases (5). Viral acute RTI are estimated to cause 75% of acute diseases in children, and is the main reason for hospitalization worldwide (6). The annual prevalence in an otherwise healthy child is from 3 to 10 infections (7). Early and recurrent lower RTI are linked to a higher risk to develop asthma or bronchiectasis (8–10). However, bronchiectasis secondary to recurrent and severe infections alone have declined, with an increasing proportion of patients being recognized as having underlying conditions predisposing to its development (11).

Improvements in immunization programs and the wide availability of antimicrobials, have led to optimism for most of the devastating infectious diseases. Always without forgetting that alleviation of poverty is crucial, the combination of genetic versatility and ecological opportunism of the microbial world appears to have been under-estimated (12). Some emerging pathogens, such as *Legionella*, avian influenza, and coronavirus species were described in the past decades (13). Ethnic variations in the incidence of RTI have also been reported, suggesting genetic susceptibility to disease (14). Most children, on reaching 2 years of age, have been in contact with the most common respiratory viruses, such as respiratory syncytial virus (RSV), but while some develop a mild disease, others develop severe bronchiolitis (15). Influenza viruses cause mild to moderate respiratory illness in most people, but some develop fatal infections. The virulence factors encoded by viral genes can explain seasonal or geographical differences at a population level, but are unlikely to account for inter-individual clinical variability (16). TB outcome depends on the pathogen and extrinsic elements, as well as on host factors that are still unclear (17).

As regards bacteria, focusing on those species whose normal ecological niche is the airways, therapeutic decisions are a daily clinical challenge (18). The shift from commensalism to infection is shaped by host intrinsic (genetics) and extrinsic factors (for example, diet and exposure to cigarette smoke and environmental pollution) and by bacterial features that also contribute to inter-individual variability (19). Bacteria develop adaptive mechanisms (at genetic/phenotypic level) in order to survive in a hostile environment, such as the respiratory tract (20, 21). Whether pathogen virulence generates clinical symptoms depends on how well the immune system limits its impact. Recently, changes in gut and lung microbiome composition (dysbiosis) have also been related to dysfunctional immune modulation (22).

## Immune Response to Respiratory Tract Infections

Respiratory immune responses are complex, and inborn errors can be present at any level. Essential pathways can be summarized as follows: Firstly, the pathogen has to be detected by host cells. This identification relies on a set of pathogen associated molecular profiles that bind to pattern recognition receptors (PRR). PRR can be found as transmembrane, cytosolic or extracellular components. Among PRRs, it is important to mention toll-like receptors (TLR), nucleotide-binding oligomerization domain-containing (NOD) receptor, NOD-like receptors (NLR), RIG-I-Like Receptors (RLR), and receptor CD14 because of their importance during respiratory infections (23). Depending on the PRR, different intracellular signaling pathways are activated (24). Most of the signaling pathways converge on signaling hubs, such as transcription nuclear factor κβ (NF-κβ), interferon regulatory factor families (IRF3, IRF7), and mitogen-activated protein kinase, leading to the induction of gene expression encoding adhesion molecules, pro-inflammatory cytokines, chemokines, and type I interferon, among others. NLRs directly trigger inflammasome assembly and caspase-1 activation, leading to interleukin (IL)-1β and IL-18 processing (25). Type III interferons, also termed IFN-λ, have been recently identified as regulators of immunity and homeostasis in the respiratory tract (26) during infections, as well as during chronic lung diseases, such as asthma and chronic obstructive pulmonary disease (COPD) (27). Alveolar macrophages and dendritic cells (DC) have an important role sensing microbes and thus activating lung epithelial cells and neutrophils. These are essential for the defense against bacteria, viruses, and *Aspergillus* (28, 29), as well as in the pathogenesis of acute lung injury. In a recent study, patterns of differentially expressed cellular genes shared by several respiratory pathogens were searched using transcriptomics (30). Most of the commonly up-regulated host genes were related to the innate immune response and/or apoptosis, with Toll-like, RIG-I-like, and NLR among the top 10 signalers. Some of the genes showed a high degree of interconnection and possible redundancy to respiratory viral and bacterial infections. The adaptive immune response requires the activation of antigen-specific T and B lymphocytes to trigger protective cellular and humoral responses. Most of the T lymphocyte subsets, along with B lymphocytes and DC, are essential for immune defense and/or regulation (31). In particular, the protective immunity against *M. tuberculosis* depends on CD4^+^ T-helper1 lymphocytes that mainly secrete interferon-gamma (IFN-γ), IL-2, and tumor necrosis factor alpha (TNF-α), which leads to macrophage activation, cytokine production, and bacterial control (32). HIV-revealed T-cell lymphopenia as a well-defined risk group for *Pneumocystis jirovecii* pneumonia (PJP), but also in other situations where CD4 lymphocyte count is lower, such as renal transplant recipients (33).

## Genetic Susceptibility to Respiratory Tract Infections

The study of susceptibility to lower respiratory tract infections is complex, and requires different approaches. There are three main elements playing a role: host genetic background (in relation to lung tissue functionality and immune response), pathogen virulence determinants, and environmental factors.

Early life (children under 5 years of age) is a challenging period because pulmonary tissue and the immune system are still in a maturation process while being continuously exposed to airborne antigens (34). However, the occurrence of life-threatening bacterial/viral/fungal infection in an otherwise healthy individual deserves further immunological and genetic studies (35, 36). Complications during upper RTI include sinusitis and otitis media, and in the lower airways, pneumonia, bronchitis, as well as the development of bronchiectasis, interstitial lung diseases, organizing pneumonia, and hyperreactive airway diseases (37). Indeed, genetic susceptibility for the concomitant illnesses that predispose to RTI can also play a role, including congenital defects of the airways, familial congenital bronchiectasis or tracheobronchomegaly (11). As regards impaired mucociliary clearance, cystic fibrosis is the most common autosomal recessive disorder and primary cause of bronchiectasis in the developed world. Mutations are well-defined, but its severity is influenced by genes involving inflammatory and anti-inflammatory mediators (38, 39). Other disorders include ciliopathies and disorders of humoral immunity. Alpha 1-antitrypsin is a circulating serine protease inhibitor (serpin) made in the liver that plays an important role in modulating immunity, inflammation, apoptosis, and possibly cellular senescence programs and its deficiency is considered the genetic cause of COPD, but there are other genetic factors that may affect disease activity and outcomes, even in patients without this deficiency (27).

High-throughput whole genome sequencing technologies and novel bioinformatics tools are revealing the sequence and annotation of the complete human genome, as well as genome-wide maps of polymorphic microsatellite markers and single nucleotide polymorphisms (SNP). In order to characterize genetic susceptibility, two complementary approaches can be envisaged: whole genome association studies (WGAS) for the identification of variants with high population frequency but low impact at individual level in terms of risk of infection (although SNP identification can potentially be later included in healthcare planning protocols); and mechanistic studies for identifying disease-causing mutations with deleterious effects, related to a high risk of infection at individual level, although its frequency in general population is low. Many genetic variants have been associated with complex human diseases and traits, but often confer relatively small increases in risk (40). According to a recent review, there are more than 300 primary immunodeficiency disorders (PIDs), most of them monogenic conditions with Mendelian inheritance, that are mainly associated with crucial defects in adaptive immunity (31). Innate immune responses are largely redundant, with pleiotropic nature of some gene products (31), thus most of the defects can be potentially counterbalanced. According to the literature, there is another view suggesting that while patients with broad immunodeficiencies may present with one of their many infections, the phenotype of particular inborn errors of immunity is very narrow, with susceptibility to only one specific infection (36, 41, 42). A set of inborn errors affecting “primarily” innate immunity, exercise their effect on the adaptive immune response (41). The range and nature of infections depend on several factors. The improving recognition of immune dysregulation diseases, autoinflammatory disorders, and interferonopathies leads to changes in terminology. The annual report of the authoritative International Union of Immunological Societies (43) has categorized and listed (as of February 2017) 354 inborn errors of immunity, and those with a predominant RTI phenotype have been included in Table 1.

**Table 1:** Reported risk of infection and recommended prophylaxis according to functional classification of biologicals-based on ESCMID consensus document (44) and to categorization of inborn errors of immunity-based on International Union of Immunological Societies annual report (43).

	**Reported risk of infection**	**Recommendations**			
		**TB screening and prophylaxis**	**Pneumocystis prophylaxis**	**Pneumococcal/capsulated bacteria vaccination**	**Influenza vaccination**
**TARGETED AGENT**					
Anti-tumor necrosis factor-α agents	Two to four increase in the risk of active TB compared to healthy patients and other granulomatous conditions Rate of *Legionella* 37-fold higher. Histoplasmosis and coccidioidomycosis	Yes	No	Yes	Yes
Interleukins, immunoglobulins and complement factors	IL-1 family, moderate risk of infection, IL-6 and IL-6 receptor (JAK), similar to TNF. Neutropenia in some cases C5 targeted, *Aspergillus* encapsulated bacteria, specially *Neisseria*, IL-17 upper respiratory tract infections and *Candida* IgE helminth infection, *Strongyloides*	Yes	No	Yes	Yes
Cell surface receptors/associated signaling pathways	Drug induced neutropenia Skin and soft tissue infections and sometimes pneumonia Overall risk of infection low for epidermal growth factor	Optional	No	Age appropriate	Yes
Intracellular signaling pathways	Increased overall risk of infection, cytomegalovirus and hepatitis B reactivation Difficult to distinguish from the risk of the underlying disease Cases of *Pneumocystis*, invasive fungal infection nocardiosis, mainly JAK	Yes	Yes	Age appropriate	Yes
Lymphoid cells surface antigens (CD19, CD20, CD52)	Can cause IgG hypogammaglobulinaemia and neutropenia Evidence of catheter related bacteremia, severe respiratory tract infection, hepatitis B reactivation and varicella zoster	Yes	Yes	Age appropriate	Yes
Lymphoid/Myeloid cells surface antigens (CD22, CD30, CD33, CD38, CD40, SLAMF-7, CCR4)	Similar to anti CD20	Optional	Yes	Age appropriate	Yes
Immune checkpoint inhibitors, cell adhesion inhibitors, sphingosine-1-phosphate receptor modulators and proteasome inhibitors	Associated T cell lymphopenia but no opportunistic infections reported Risk of varicella zoster virus	Yes	Yes	Yes	Yes
**INBORN ERRORS OF IMMUNITY**					
Severe combined immune deficiency:	Severe opportunistic disseminated infections in early childhood	Non-applicable	Yes	No	No (cohabitants)
Less severe combined immune deficiency	Some related to recurrent respiratory tract infections	Optional	Yes	Yes	Yes
Combined immune deficiencies with syndromic features e.g., Wiscot Aldrich and those altering DNA reparation	Recurrent infections	Optional	No	Yes	Yes
Humoral immune deficiencies Antibody deficiencies	Repeated respiratory tract infections (pneumonia, sinusitis, otitis, …)	Optional	No	Yes	Yes
Defects of phagocyte number or function	Fungal and bacterial infections, pulmonary abscesses, aspergillosis	No	No	Yes	Yes
Defects in intrinsic and innate immunity	Pyogenic bacterial infections	In selected cases	No (^*^)	Yes	Yes
Autoinflammatory diseases	No clear predisposition to infection	No	No	Yes	Yes
Complement deficiencies	Disseminated infections (meningitis/sepsis) by capsulated microorganisms and *Neisseria*	No	No (^*^)	Yes	Yes

* *Antibiotic prophylaxis to prevent bacterial infections*.

Despite the limitations of molecular genetic studies in pulmonary infections, several associations have been described between SNPs and bacterial pneumonia and mycobacterial infections (14, 45). Polymorphisms affecting community-acquired pneumonia including, among others, those related to mannose-binding lectin and the IgG2 Fc gamma receptor II, and are discussed extensively elsewhere (14). The genetic contribution for the propensity to develop severe RSV infection was estimated to account for ~20% of the variance in RSV disease severity. Several studies have attempted to link candidate host SNPs to disease severity, mostly in chemokine receptors and PRRs (46, 47).

As regards TB, SNPs are frequently found in loci involving TLR-2, TNF-α, IL-12, and IFN-γ, and their corresponding receptors (45). Genetic variations in dendritic cell-specific ICAM-3 grabbing non-integrin have been linked with reduced risk of developing TB (48). Mendelian susceptibility to mycobacterial disease is a syndrome characterized by susceptibility to weakly virulent mycobacteria, including the attenuated vaccine Bacillus Calmette-Guerin (BCG) strain and non-tuberculous mycobacteria (NTM). Different gene mutations have been identified, most of which are related to IFN-γ-mediated immunity (49–51). Using exome and transcriptome sequencing, three rare loss-of-function variants have been recently characterized in theIFIH1 gene. These encode a RIG-I-like receptor involved in the sensing of viral RNA (52). The deficiency causes a primary immunodeficiency manifested in extreme susceptibility to common respiratory RNA viruses. Interestingly, human primary immunodeficiency disorders (PID) affecting T and B cells were not found to predispose to severe influenza. However, human IRF7 was shown to be essential for IFN-α/β- and IFN-λ-dependent protective immunity against primary influenza *in vivo* (53).

## Impact of Biological Response Modifiers in Respiratory Tract Infections and Tuberculosis

Biological response modifiers (BRM) are substances that interact with and modify the host immune system by acting on a therapeutic target considered important in the pathogenic process of the disease. Monoclonal antibodies (mAbs) are now established as therapies for malignancies, transplant rejection, several immune disorders from most organ systems, and even infectious diseases (54). Safety problems related to immunomodulation and infection have been identified in some cases (55). The use of mAb indirectly provides insights into the function of the molecule to combat particular pathogens, increasing our knowledge of the immune system (56). A recent consensus document has reviewed the groups of drugs according to the targeted site of action, the expected impact on susceptibility to infection, the evidence of risk, and the recommendation of prevention strategies. It is also important to mention the influence of previous or concomitant therapies, underlying conditions, and the accumulative exposure to the agent (44). As regards lower RTI, treatment with BRM results in an increased risk is reported for pneumonia, influenza-related complications, TB and NTM, *Pneumocystis*, and fungal infections, such as histoplasmosis, taking into account the impact of geographical variations on incidence rates (57). The knowledge obtained from experience with the prescription of BRM may be particularly valuable for the understanding of some genetic inborn errors, as the type of infections acquired as a side effect may help to identify which genetic defects favors a similar infectious phenotype. With the current knowledge and because of pleiotropic effects, it is not feasible to show how biological agents actually mimic some inborn errors of immunity, but several parallelisms can be inferred. We provide a Table containing the list of BRM according to their functional classification, and inborn errors categorized according to common infectious phenotypes (Table 1). Data presented are extracted from the respective consensus documents, and lists the main RTI and preventive recommendations.

**Figure 1:**
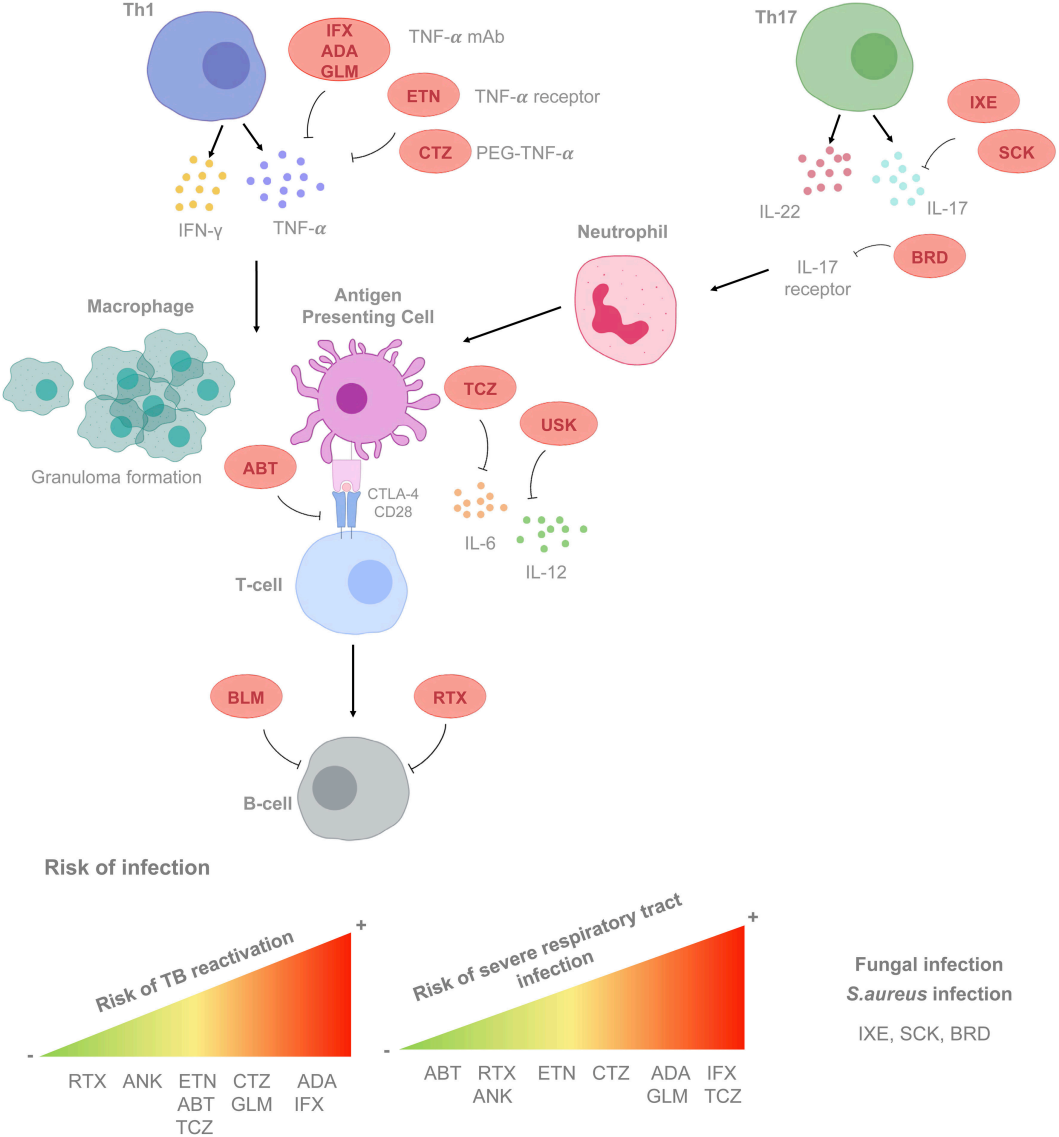
Mode of action of biological response modifiers (BRM) according to cell type, cytokine and/or receptor targeted. Risk of developing infections according to the BRM considered is also shown. List of BRM. Anti-tumor necrosis factor-α (TNF-α) agents. ADA, adalimumab; CTZ, certolizumab; GLM, golimumab; IFX, infliximab; ETN, etanercept. Anti-interleukins, immunoglobulins, and complement factors. Anti IL-1, anakinra ANK; Anti IL-6: TCZ, tocilizumab; Anti IL-17: SCK, secukinumab; IXE, ixekizumab; BRD, brodalumab. Anti-IL12/23: USK, ustekinumab. Cell surface receptors/associated signaling pathways agents. Anti-CD28: ABT, abatacept; B-cell activating factor (BAFF): BLM, belimumab. Lymphoid cells surface antigens. Anti-CD20: RTX, rituximab. mAb, monoclonal antibody; PEG, polyethylene glycol; TB, tuberculosis; LTBI, latent tuberculosis infection.

Current recommendations should be focused on rheumatic diseases because of the greater experience in follow-up time (more than 15 years) and number of patients treated. Biological therapies targeting TNF-α, T cells, B cells, and various cytokines (including IL-6 and IL-1) have become essential for the treatment of rheumatic diseases [mainly rheumatoid arthritis (RA), ankylosing spondylitis, and psoriatic arthritis], as well as other immune-mediated diseases. Moreover, additional drugs with novel targets, including those that inhibit IL-12–IL-23, IL-17α, or the Janus activating kinase system have been introduced more recently. Immunomodulation offered by biological and non-biological disease-modifying therapies and prednisone contributes greatly to the increased risks of opportunistic infections (OI) (58, 59). In Figure 1 we present the sites of action and associated risks of the most frequently prescribed BRM.

Two recent meta-analysis have calculated the relative risk of infection for rheumatic patients under biological treatment, with an odds ratio (OR) of 1.31–1.41 (60, 61). The absolute increase in the number of serious infections per 1,000 patients treated/year is six times higher than that observed with synthetic disease-modifying anti-rheumatic drugs (DMARDs). Different meta-analyses and national registries have confirmed the increase on the impact of any infections (20%), serious infections (40%), and TB (250%), associated with anti-TNF-α use (60). In addition, the risk of serious infections is highest during the first 6 months of therapy (62) (up to 4.5-fold risk), although, after 1 year this risk is no different from conventional DMARDs. Recurrent infections in RA are common. In a prospective observational cohort study, the baseline annual rate of a first serious infection was 4.6%. Additionally, 14% of this cohort experienced a recurrent episode/year during their follow-up, with the highest risk being within the first year (29%), and with respiratory infections being the most common (44% of all episodes) (63). Factors that have shown to be predictive of infection include, age, functional status, specific comorbidities (chronic renal/lung disease), corticosteroid treatment, number of previous DMARD, treatment failures, previous serious infections, and current treatment with anti-TNF-α inhibitors or non-biological DMARDs (64). Nevertheless, recent data suggest that patients having a serious infection and exposed to biological treatment have a significantly lower risk of sepsis and fatal outcome than patients treated with conventional DMARDs (62, 65). British and French national biological registries have reported OI rates of 200–270/100,000 in patients using anti-TNF-α therapies (66, 67). In particular, there is evidence of an increased risk of *M. tuberculosis*, herpes zoster, and *Listeria* infections. The overall incidence of OI is not significantly different considering drug classes; however, the rate of PJP is significantly higher in those patients using rituximab in comparison to anti-TNF-α therapy. The absolute risk of PJP is low, although corticosteroid exposure is a strong predictor. Current data do not support PJP prophylaxis for all rituximab users. However, it may be appropriate in certain high-risk individuals. Furthermore, rituximab-associated neutropenia and impaired antibody response is also well-described.

Pre-clinical and clinical evidence indicate that anti-TNF-α therapy (infliximab, adalimumab, golimumab, certolizumab pegol, and etanercept) is associated with a 2- to 4-fold increase in the risk of active tuberculosis and other granulomatous conditions. Risk seems to be lower for etanercept (68). Risk also depends on local TB prevalence: in the year 2000, Spanish investigators reported an estimated TB incidence of 1,893/100,000 person-years in patients with RA treated with infliximab (69). This rate is ~10- to 20-fold higher than the observed rate in naïve patients. These rates have decreased dramatically since the establishment of latent tuberculosis infection (LTBI) screening prior to biological therapy (67, 70). It is essential to rule out LTBI in such individuals in order to reduce the risk of active TB reactivation. Interferon-gamma release assays (IGRAs) are useful tools for LTBI diagnosis. They are more specific than the tuberculin skin test (TST) because they do not show cross-reactivity with BCG-vaccination or NTM sensitization (71–73). Moreover, these *in-vitro* assays incorporate a mitogen control that can detect the presence of anergy, common in patients on immunosuppressive therapy (74). However, the clinical performance of IGRAs is still controversial due to the variety of concomitant immunosuppressive drug-regimens used at the time of LTBI screening, population heterogeneity, and the severity of the disease itself (75). Therefore, the clinical accuracy of IGRAs seems to be differentially affected depending on the specific type of immune disorder. Crohn's disease and/or its concomitant drug-profile (such as azathioprine or high-dose corticosteroids) could negatively affect the clinical performance of IGRAs when compared with other immune-mediated diseases, such as psoriasis or inflammatory rheumatic diseases (76). Thus, it seems prudent and convenient to perform dual LTBI testing with TST and IGRAs (77). Patients with RA and underlying structural lung diseases are at increased risk of developing NTM infection (78), mostly *Mycobacterium avium*. In some countries, NTM infections are more common than TB after anti-TNF-α treatment. However, there are still no established recommendations as regards screening and prophylaxis (79). A baseline chest x-ray should be recommended prior to starting therapy, and in patients with chronic unexplained cough, further work-up should include chest computed tomography scans and culture of respiratory specimens.

Immunization strategies are recommended for all cases, regardless of whether the patient has PID or is receiving immunosuppressive treatment, and it is of importance to be vaccinated according to the national immunization routine schedules. For patients with anti-TNF-α treatment, pneumococcal and age-appropriate anti-viral vaccinations (i.e., influenza) should be administered (68). Immunization before and after BRM is well-established as regards inactivated vaccines, and precautions should be taken for live vaccines (57). However, even if response to vaccines is impaired in patients with PID (80), it may have an effect in patients receiving some BRM. This may be partially explained by the concept of trained immunity-based vaccines (81).

In conclusion, RTIs belong to the most common causes of infections in humans worldwide. The genetic contribution to severe RTIs may have been masked by other interventions (82). The inborn errors of innate immunity show us that the absence of a measurable immunological defect does not exclude an immunodeficiency (41). Further functional genetic studies are necessary in order to fully validate the impact of host genetics during lung infections. The knowledge obtained from experience with the prescription of BRM may be particularly valuable, as the infections acquired as a side effect may help to identify genetic defects with a similar infectious phenotype. In the meantime, recommendations based on biological rationale and clinical experience are mandatory in order to prevent re-emerging severe infections.

## Author Contributions

CP organized the structure and supervised the manuscript elaboration, revised literature and wrote a part of every chapter. AL revised literature, wrote the sections related to the immune response to infection and part of genetics, and edited the manuscript. IL, RV-H, and JD revised and wrote the aspects related to tuberculosis, and JD also supervised the manuscript elaboration. LM revised and wrote the section regarding the impact of biological response modifiers specially related to rheumatologic diseases. LM, AL, and RV-H prepared the figure. MM and CR revised aspects related to immunodeficiencies, and impact of BRM in children. IB revised host genetic factors. All authors revised and approved the final version of the manuscript.

### Conflict of Interest Statement

The authors declare that the research was conducted in the absence of any commercial or financial relationships that could be construed as a potential conflict of interest.
